# Datawiz-IN: Summer Research Experience for Health Data Science Training

**DOI:** 10.21203/rs.3.rs-4132507/v1

**Published:** 2024-03-29

**Authors:** Sadia Afreen, Alexander Krohannon, Saptarshi Purkayastha, Sarath Chandra Janga

**Affiliations:** 1 Department of BioHealth Informatics, Indiana University - Purdue University Indianapolis, Indianapolis, 46202, IN, USA.

**Keywords:** AI Diversity, Equity, Inclusion, Data Science Training, Biomedical Education, Summer Research Experience

## Abstract

**Background:**

Good science necessitates diverse perspectives to guide its progress. This study introduces Datawiz-IN, an educational initiative that fosters diversity and inclusion in AI skills training and research. Supported by a National Institutes of Health R25 grant from the National Library of Medicine, Datawiz-IN provided a comprehensive data science and machine learning research experience to students from underrepresented minority groups in medicine and computing.

**Methods:**

The program evaluation triangulated quantitative and qualitative data to measure representation, innovation, and experience. Diversity gains were quantified using demographic data analysis. Computational projects were systematically reviewed for research productivity. A mixed-methods survey gauged participant perspectives on skills gained, support quality, challenges faced, and overall sentiments.

**Results:**

The first cohort of 14 students in Summer 2023 demonstrated quantifiable increases in representation, with greater participation of women and minorities, evidencing the efficacy of proactive efforts to engage talent typically excluded from these fields. The student interns conducted innovative projects that elucidated disease mechanisms, enhanced clinical decision support systems, and analyzed health disparities.

**Conclusion:**

By illustrating how purposeful inclusion catalyzes innovation, Datawiz-IN offers a model for developing AI systems and research that reflect true diversity. Realizing the full societal benefits of AI requires sustaining pathways for historically excluded voices to help shape the field.

## 1 Introduction

Artificial intelligence (AI) adoption is rapidly expanding across sectors, yet equitable access and outcomes remain elusive [1]. However, concerns exist that AI systems may unintentionally perpetuate biases, yielding inequitable outcomes [2]. Diverse perspectives must inform AI ethics and governance to mitigate such risks. Currently, AI guidelines and regulations disproportionately reflect the viewpoints of industrialized nations, failing to account for the distinct values of less developed regions [3, 4]. Homogeneous perspectives embedded in many AI systems risk perpetuating biases and limiting societal benefits [1, 5]. Accordingly, emerging research underscores the need to incorporate diverse viewpoints, especially those from historically marginalized groups, to ensure just AI progress, including in education [6].

**Fig. 1:**
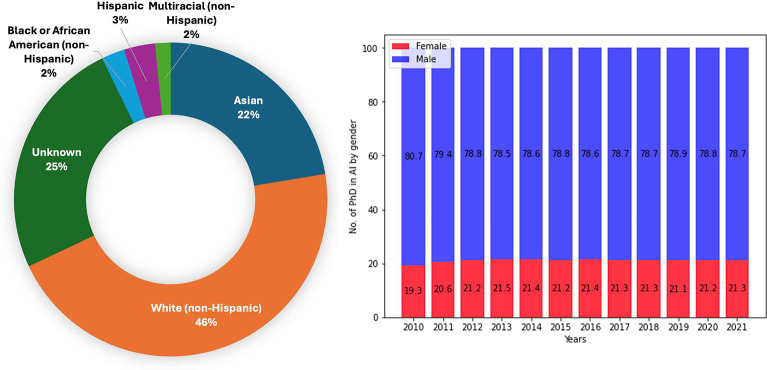
Race-Ethnicity of PhD students in AI [13]

Currently, women and racial minorities are significantly underrepresented in the AI field [7], reflecting longstanding diversity gaps in technology-related disciplines [8] (See Figure 1). Recognizing these disparities is essential, as diversity spurs innovation, corrects biases, and promotes user-centric design [9]. Proactive efforts to increase participation across gender, ethnic, and academic backgrounds can help realize these tangible benefits.

Though industrialized nations have dominated AI research, deployment across geographic regions makes inclusion an imperative [10]. Currently, representation of women and racial minorities remains as low as 10–15% in the AI field [11]. Realizing the tangible benefits of diversity, including spurring innovation, correcting bias, and boosting user-centric designs, requires proactive efforts [12].

We introduce Datawiz-IN, an NIH-funded initiative designed to increase the participation of students from marginalized backgrounds in AI healthcare skills training through immersive summer research experiences. In alignment with Indiana University’s broader efforts to expand access through programs such as IU-MSI, Datawiz-IN offers comprehensive educational opportunities with an explicit focus on applied AI techniques. This summer-long, immersive program spans data science, machine learning, and computational methods, equipping students with the tools necessary to drive healthcare projects leveraging AI. Through assertive outreach to women and minority groups, Datawiz-IN has achieved a level of representation that truly reflects diversity.

### 1.1 Our Contribution:

Several related programs share similar goals to Datawiz-IN, each offering unique approaches to promote diversity and inclusion in AI. AI4ALL, for example, provides AI training to underrepresented groups and has achieved a notable 40% participation rate from women [14]. Industry initiatives, such as Google’s Bias-busting @ Work and IBM’s diversity in AI programs, also strive to foster inclusion. However, our program distinguishes itself by focusing specifically on health data science and AI, leveraging existing partnerships, such as the Indiana University-Minority Serving Institutions (IU-MSI), for recruitment purposes. Moreover, we provide cohort-based mentoring and support to enhance STEM identity among our trainees, utilizing a near-peer and role model approach [15] to guide students through their summer experiences.

In the following sections, we outline our strategies for improving a ”sense of belonging” among undergraduate students, which include fostering inclusive environments, implementing mentorship programs, establishing affinity groups, employing mindset interventions, and promoting growth mindset training.

## 2 Background

### 2.1 Institutional Commitment to Diversity (IU-MSI STEM Initiative)

The IU-MSI STEM Initiative fosters collaboration between Indiana University and partnering institutions to advance science, technology, engineering, and mathematics (STEM) education and research. The initiative focuses on four interconnected goals: increasing minority representation in graduate and professional ranks, enabling faculty research partnerships across diverse universities to broaden participation, leveraging external funding opportunities to strengthen collaborations and accelerate discovery, and promoting supportive structures, such as faculty exchanges, to enrich joint proposal development, networking, and teaching. These pillars work harmoniously to cultivate symbiotic relationships, progressively dismantling barriers faced by historically underrepresented groups and institutions through intentional access and dialogue.

#### Evolution of the IU-MSI STEM Initiative:

Originating in 2006, the IU-MSI STEM Initiative was born with the express vision to spur minority representation and leadership in scientific fields. The early successes in fostering inclusion through collaborative programs paved the way for subsequent interventions, such as *Datawiz-IN*, to build upon this foundation. By 2009, the initiative had established relationships with 12 Historically Black Colleges and Universities (HBCUs) and launched summer research programs. In 2015, the initiative received a significant boost through major Department of Navy funding, allowing its scope to expand beyond HBCUs to include Hispanic-serving institutions and Tribal Colleges. This widened collaboration led to a fitting rebranding as the Minority Serving Institution STEM Initiative [16].

#### 2.1.1 Key Achievements:

**Fig. 2:**
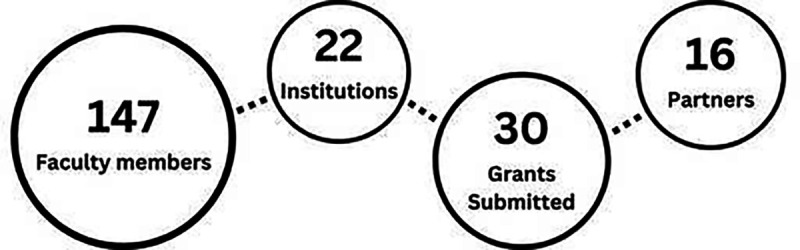
Milestones of IU-MSI. [16]

The expanded cross-institutional participation reinforced the university’s commitment to diversity by enabling faculty members across minority-serving institutions to advance innovation collaboratively (see Figure 2). The growth of these networks strengthened exchanges centered around research, proposal development, and teaching, ultimately unlocking student potential. The renewed emphasis on access and collaboration continues to erode systemic barriers, creating a more inclusive and equitable environment for all.

Indiana University’s strategic investments in cross-institutional platforms, such as Datawiz-IN, align with broader national priorities aimed at enhancing diversity in STEM fields. This momentum is exemplified by the NIH R25 initiative, which directly addresses the urgent need to promote diversity by providing grants for specialized training programs in biomedical informatics and data science. According to the National Library of Medicine (NLM) (2023), R25 programs actively seek to increase the representation of historically marginalized groups in graduate programs and research careers related to impactful new technologies.

### 2.2 NIH R25 Goals and Vision

**Fig. 3:**
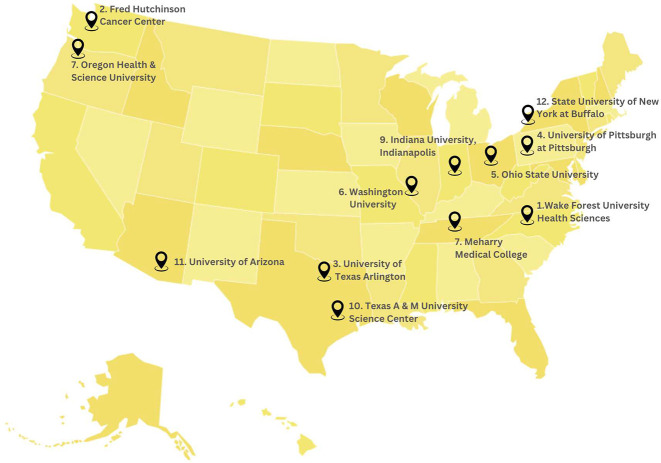
Spread of NLM R25 initiative across various institutions

The NIH R25 initiative mirrors the diversity priorities catalyzed by the effective partnerships established through the IU-MSI STEM Initiative. By providing targeted grants, the R25 initiative confronts marginalization in biomedicine, financing skills training for historically excluded groups in burgeoning data science fields. As outlined by the NLM (2019), R25 programs actively strive to rectify representation gaps and empower impactful research conducted by students from underrepresented backgrounds. Prior to examining Datawiz-IN as an R25-funded case study model, it is essential to outline the motivations and high-level goals guiding the broader R25 initiative, which helped prime Indiana University to secure such directed funding. Launched by the NIH, these research education grants support hands-on training programs that develop specialized expertise rather than broad frameworks, allowing for tailored upskilling interventions that respond to pressing needs. By enabling research training collaborations at over 12 higher education institutions (Figure 3), the R25 initiative paved the way for partnerships like Datawiz-IN to confront systemic marginalization. [17].

### 2.3 Objectives for Datawiz-IN training program:

The R25 program’s overarching goal is to expand the representation and preparation of historically excluded groups in the upper echelons of biomedicine and artificial intelligence (AI). With the support of multi-year NIH funding, Datawiz-IN has two central priorities: broadening academic and research career opportunities for marginalized students and equipping them with impactful emerging technologies, such as AI.

#### Diversifying Progression Pathways and Dismantling Barriers:

The primary aim of the R25 program focuses on diversifying progression pathways by encouraging the participation of minoritized populations in graduate programs and professorships, from which they have long been excluded [18]. In parallel, hands-on training in biomedical data science and AI methods not only builds technical capabilities but also serves to dismantle unfair barriers to access and advancement [19]. We envision that such targeted interventions will progressively chip away at the lack of diversity that restricts innovation and prevents ”leaky academic pipelines,” which further concentrate AI control and benefits in the hands of a privileged few [20].

## 3 Materials and Methods

### 3.1 Participant recruitment approaches

The DataWiz-IN program employs a comprehensive and inclusive approach to scholar selection, ensuring a diverse cohort of talented individuals who demonstrate academic excellence and a strong commitment to pursuing careers in biomedical informatics and data science. The selection criteria are designed to identify scholars with the qualifications, motivation, and potential to succeed in the program and contribute to the field. We have the following selection criteria:

1. **Eligibility criteria:** Scholars must be citizens, nationals, or lawfully admitted permanent residents of the United States.
2. **Residential requirement:** They must also commit to relocating to Indianapolis and residing in the on-campus residential-based learning community (RBLC) during the 12 weeks of summer.
3. **Economically disadvantaged preference:** Preference is given to those from economically disadvantaged backgrounds, like those receiving Pell grants or other need-based scholarships. Additionally, first-gen students are prioritized. This ensures the program supports students who may otherwise face barriers to accessing advanced education and training opportunities.
4. **Academic achievement:** DataWiz-IN scholars must have an overall GPA between 3.0 and 4.0, a range chosen to populate the applicant pool and provide a basis for selectively shaping the scholar cohort. This ensures that the program attracts high-achieving students well-prepared to engage in rigorous research and training activities.
5. **Letter of recommendation:** The DataWiz-IN program seeks scholars who demonstrate good character, motivation, and professionalism. Applicants are required to submit at least one letter of recommendation attesting to their talents and interests related to information technology.
6. **Personal statement:** Scholars must submit a personal statement of 400–500 words outlining their academic and professional goals. This statement allows applicants to articulate their interests, aspirations, and commitment to pursuing careers in biomedical informatics and data science.

These letters can highlight qualities such as passion, persistence, grit, innovation, and the ability to collaborate, work in teams, and problem-solve in project-based learning environments. The program also accepts letters from civic and lay members of the community for students who are home-schooled or attend charter schools with limited staff, ensuring that the selection process is inclusive and recognizes the diverse backgrounds and experiences of applicants. By employing these innovative and comprehensive selection criteria, the DataWiz-IN program aims to recruit a diverse and talented cohort of scholars who are well-positioned to succeed in the program and make significant contributions to the field of biomedical informatics and data science.

### 3.2 Evaluation Framework

The program outcomes were assessed through the triangulation of quantitative and qualitative data [21] across three key areas. First, diversity gains were tracked to measure the program’s success in promoting inclusion [22]. Second, project analysis was conducted to evaluate research productivity, including an assessment of methods, visualizations, and domain impacts [23]. Finally, a participant survey was administered to gauge the participants’ experiences, focusing on skills gained, support quality, challenges faced, and overall sentiments [24]. This multi-perspective evaluation approach aligned with the core programmatic goals of fostering inclusion, enabling innovation, and developing future leadership in the field.

### 3.3 Participant Data Collection

The program evaluation utilized data spanning three primary areas: representation, innovation, and experience. Representation data were obtained through participants’ self-reported demographics, including ethnicity, gender, background, and intersectional dimensions, allowing for comprehensive diversity tracking. Innovation data consisted of the computational projects completed by trainees, which applied machine learning, data science, and AI techniques. These projects were systematically reviewed to measure research productivity. Additionally, a mixed-methods survey was conducted to collect participants’ perspectives on skills gained, support quality, challenges encountered, and overall program sentiments. The survey included both numerically scored items and open-ended responses, capturing a rich set of experiential data. By combining these diverse data sources, the evaluation enabled a holistic analysis of the program’s impact on diversity, productivity, and participant experiences.

### 3.4 Data Analysis

A combination of quantitative and qualitative methods was employed to analyze program outcomes across the representation, innovation, and experience dimensions. Diversity gains were quantified through demographic data analysis, providing insights into the program’s success in promoting inclusion. Computational projects were systematically reviewed to assess research productivity, focusing on the application of cutting-edge techniques and the generation of meaningful insights. Survey responses were analyzed using a two-pronged approach. Numerically scored items were subjected to statistical analysis, while open-ended responses were analyzed using sentiment analysis techniques, specifically the *Syuzhet* R package, to assess participant attitudes and perceptions.

## 4 Results

### 4.1 Outcomes and Impact of the Program:

#### 4.1.1 Participant Diversity

**Fig. 4:**
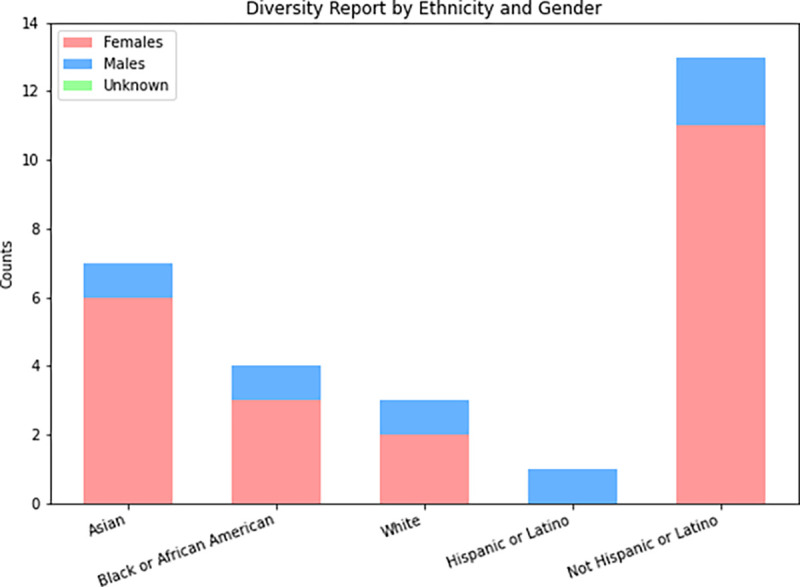
Participant diversity of the program

Datawiz-IN demonstrated a strong commitment to diversity by achieving significant representation of historically marginalized identities among its summer research interns. The program prioritized inclusion in its recruitment and enrollment processes, employing targeted outreach strategies to attract talented students from underserved groups. Figure 4 illustrates the distribution of participants across gender identities, ethnic communities, and intersectional experiences, reflecting the program’s successful efforts to promote diversity. With women and minorities each representing over 60% of the participant pool, Datawiz-IN made notable progress in advancing representation, aligning with its core values. This diverse cohort of participants contributes to the program’s robust discourse, innovation, and discovery outcomes by bringing together various perspectives and experiences.

#### 4.1.2 Innovations and Impacts: Spotlight on Participant Projects

Datawiz-IN supported the development of impactful projects across various disciplines. Interns employed a range of approaches, including gene sequencing, spatial transcriptomics, machine learning, epidemiological analysis, database reviews, and imaging techniques, to advance understanding in fields such as neuroscience, RNA biology, kidney disease, and post-COVID conditions (Table 1). Their work contributes to elucidating disease mechanisms, enhancing diagnosis and treatment, and providing insights into the representation of underserved groups.

**Fig. 5:**
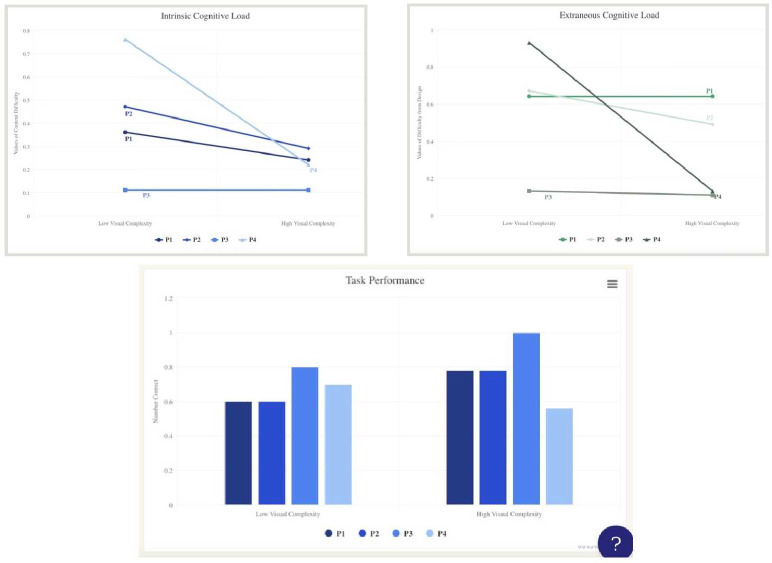
One of the Intern’s study reveals that higher visual complexity is perceived as more engaging, yet imposes a greater cognitive load, leading to reduced task performance. This inverse relationship between visual complexity and cognitive efficiency is depicted in Graphs 1 to 3, showcasing a qualitative analysis of participant feedback.

Intern projects addressed various research questions, such as identifying target brain regions for Alzheimer’s therapies, revealing novel RNA editing techniques, improving ICU delirium management, and analyzing health disparities. By providing young scholars with opportunities to apply cutting-edge methodologies within their respective domains, Datawiz-IN fostered innovative thinking and supported meaningful research with real-world implications. The program demonstrates how promoting diversity and inclusion can lead to new scientific perspectives and contribute to the discovery of solutions for pressing medical challenges. Figure 5 presents visual summaries of two selected projects, illustrating the program’s broad impact.

#### 4.1.3 Experience Evaluation

**Fig. 6:**
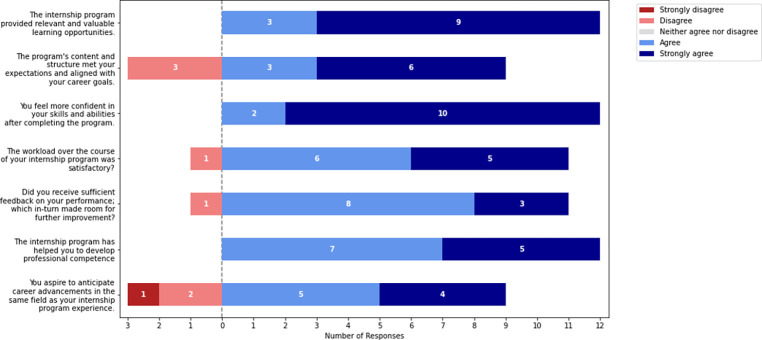
Exit survey responses on 7-point Likert scale

The exit survey contained seven 7-point Likert scale questions, two 3-point Likert scale questions and four open-ended questions that received a lot of detailed responses (see Supplementary doc). We got 85% response rate for the survey, with the 7-point likert scale responses shown in Figure 6.

The exit survey, as evidenced by participant testimonials, underscored the program’s success in providing valuable learning opportunities, with 87% of respondents expressing strong agreement. One participant remarked, ”*I loved how the fellowship was set up. Meeting so many different people helped boost my confidence*,” highlighting the diverse and enriching interactions facilitated by the program. This sentiment was further reflected in the participant confidence in their skills post-internship, with over 90% reporting significant gains. Another participant stated, ”*I gained proficiency in retrieving promoter sequences from UNIPROT... I am confident that these newly acquired skills will greatly contribute to my success and growth*,” illustrating the tangible skill enhancements and increased self-assurance among participants.

Despite these positive outcomes, one-third of the participants noted challenges related to adapting to new skills, managing time constraints, and balancing personal responsibilities. One participant candidly shared, ”*I was new to everything, so I had to learn as I went along. Although that was a bit challenging, it paid off*,” reflecting resilience and the eventual rewards of overcoming these hurdles. These reflections, summarized in Table 2, highlight both the strengths and areas for improvement within the program, illustrating a journey of growth, challenge, and confidence-building for participants.

The sentiment analysis presented in Table 2 reveals varying degrees of program effectiveness. While Overall Program Satisfaction shows a significant spread, indicating diverse experiences, the majority of participants reported positive outcomes in Skills Confidence and Valuable Learning Opportunities. However, the alignment of the Program’s Content with Career Goals received mixed reviews, suggesting a need for better customization to participant aspirations. The feedback on Developed Professional Competence indicates a positive impact, but also points to potential areas for further development. These findings underscore the program’s strengths in fostering skills and learning, particularly in the domain of AI, while emphasizing the importance of aligning content with participant goals and ensuring uniform satisfaction.

## 5 Discussion

The analysis of the Datawiz-IN program highlights the multifaceted benefits of prioritizing diversity and inclusion in AI education and research. The program’s success in achieving substantial representation gains, with four times more female and minority participation in the first cohort, challenges the outdated notion that excellence and equity are mutually exclusive. The projects’ promising innovations, spanning disease mechanisms, clinical tools, and health disparities, further demonstrate the potential of diverse perspectives in driving meaningful advancements.

Through the experience of one summer in the Datawiz-IN program, several key strategies emerged that can enhance the sense of belonging among undergraduate students in health data science:

1. Fostering inclusive environments: Creating welcoming spaces for peer interactions and communication has been shown to positively impact belonging for marginalized STEM students [25]. Implementing inclusive language and signage, while encouraging respectful dialogue and active listening, can contribute to a more supportive and inclusive atmosphere.
2. Role model mentors: Mentoring plays a crucial role in connecting students with role models who share similar identities and experiences, thereby enhancing their sense of belonging [26]. Formal university mentor schemes that pair undergraduate students with senior STEM students or faculty members can provide valuable academic guidance and psychosocial support, nurturing a sense of community and belonging.
3. Affinity groups through cohort-based activities: Cohort-based activities also proved effective in promoting a sense of belonging among Datawiz-IN participants. Engaging in social activities such as visiting the Indianapolis Canal, exploring neighboring parks, and sharing cultural experiences through food-sharing hotpot lunches fostered a strong sense of camaraderie and cultural exchange, ultimately enhancing the students’ sense of belonging within the program.
4. Mindset interventions: Brief activities addressing belonging uncertainty and stereotype threats have shown promise in improving persistence for women and minorities in STEM programs [27]. Reframing struggles as events that are common and surmountable boosts motivation and achievement.
5. Growth mindset training: Workshops training students to see intellectual abilities as malleable through effort have been shown to protect students’ sense of belonging when facing setbacks or criticism [28]. This promotes resilience, particularly among negatively stereotyped groups.

While the 14-student scope of Datawiz-IN limits its current generalizability, the intersections between social identities that layer marginalization present complexities warranting more nuanced analysis. Holistic longitudinal tracking of outcomes, akin to other NIH-funded programs [29], will be essential for gauging the program’s effectiveness and revealing individual and group impacts.

The wider implementation of similar programs faces systemic barriers, such as bias and microaggressions pervasive in many campus climates. Tailored programming and resources are needed to mitigate these obstacles across enrollment, matriculation, and career stages, as exemplified by other IUPUI initiatives [30]. Datawiz-IN serves as a model for productive pathways institutions can adopt to foster inclusion and innovation. Its multifaceted assessment framework enables continuous improvement while directing resources toward the participation, creativity, and advancement of marginalized populations in AI.

As AI’s influence rapidly expands, accelerating efforts to promote diversity and inclusion remains imperative. Equal participation in shaping emerging solutions is essential to prevent cementing inequality and to harness the full potential of diverse perspectives in driving innovation and applications that benefit society as a whole. This relies on education and industry steadfastly welcoming marginalized groups at every career level to guide progress.

While promising pilots like Datawiz-IN warrant scaling, inclusion indicators in upper ranks will serve as a litmus test for the impact of the program’s vision on reality. Creative projects showcasing the contributions of marginalized teams reveal the valuable assets accrued from diversity. Pursuing input parity alongside sustained evaluative rigor can help realize the potential of data science fields in advancing research and technology for humanity’s collective advancement.

## 6 Conclusion

The analysis of the Datawiz-IN program provides compelling evidence for the benefits of prioritizing diversity and inclusion in AI research and education. The program’s success in fostering quantitative representation gains and qualitative innovation demonstrates the untapped potential of equitable access in driving meaningful advancements. By creating pathways for equal participation, Datawiz-IN exemplifies a model for developing AI systems and research that reflect the diversity of our society.

As AI continues to permeate various aspects of our lives, it is crucial to ensure that the development and deployment of these technologies are guided by diverse perspectives. Failing to do so risks perpetuating and even exacerbating existing inequalities. Conversely, by actively promoting inclusivity and equal participation, we can harness the full potential of AI to address societal challenges and drive innovation that benefits all of humanity.

The Datawiz-IN program serves as a blueprint for cultivating inclusion and excellence in AI research and education. Its multifaceted approach, combining targeted outreach, supportive mentorship, and immersive research experiences, has proven effective in engaging and empowering students from historically marginalized backgrounds. The program’s emphasis on fostering a sense of belonging through inclusive environments, peer support, and mindset interventions highlights the importance of addressing the psychosocial factors that can impact student success and persistence in STEM fields.

## Figures and Tables

**Table 1:** 2023 Datawiz-IN Project

Interns	Project methods and approach	Undergrad Degree	Home Institution
Intern 1	Gene sequence retrieval from NCBI, upstream analysis via UNIPROT, and motif identification using MEME Suite.	MS Bioinformatics	IUPUI, Indianapolis, IN
Intern 2	Data integration from CDC and NIH, population data from Census Bureau, with analysis via Python and visualization using histograms, pie charts, and Seaborn heatmaps.	BS Biology	North Carolina Agricultural and Technical State University, Kernersville, NC
Intern 3	A pilot study on visual complexity’s effect on cognitive engagement and planned a follow-up for cognitive load in TBI patients.	BA Psychology	University of South Florida, St. Petersburg, FL
Intern 4	Used machine learning to assess Marion County’s SIDS rates during COVID-19, analyzing social vulnerability, race, and eviction data.	BS Biomedical Informatics	IUPUI, Indianapolis, IN
Intern 5	Reviewed 15 RNA modification databases for species representation, biotypes, accessibility, and 2023 updates, including categorization methods.	BS Biology	Augustana College, Rock Island, IL
Intern 6	Studied trichostatin-a’s brain impact for AD using gene data from LINCS L1000 and spatial transcriptomics, to pinpoint treatment-affected regions.	BS Medical Sciences	University of Cincinnati College of Medicine, Cincinnati, OH
Intern 7	Analyzed CRISPR Cas13 RNA editing in HEK293 cells using RNA-seq, highlighting data quality importance in CRISPR research.	BS Biology	Universidad Ana G. Mendez, Carolina, Puerto Rico
Intern 8	Examined delirium biomarkers in ICU patients versus healthy individuals, focusing on muscle and brain indicators, to enhance understanding and treatment approaches.	BS Biology	Xavier University Of Louisiana, Thibodaux, LA
Intern 9	Applied machine learning and MRI data analysis to assess BrainAGE as a biomarker for brain aging, linking it to cognitive decline and LMCI risk.	BS Chemistry	Purdue University, West Lafayette, IN
Intern 10	Analyzed kidney cellular neighborhoods in CKD and AKI patients using CODEX imaging and Fiji/ImageJ software, to discern structural disease differences.	BS Biology	Indiana University, Bloomington, IN
Intern 11	Utilized machine learning and XGBoost for feature selection and impact analysis to identify post-COVID condition patients from EHR data, referencing a rules-based phenotype.	BS Electrical Engineering	Texas A and M University, College Station, TX
Intern 12	Investigated DYRK1A ortholog mbk-1 in C.elegans for Down syndrome research, using motility assays and chemoattraction tests to understand mobility implications.	BS Public Health Studies	John Hopkins University, North Plainfield, NJ
Intern 13	Analyzed MMP roles in heart and metabolic diseases using GTeX and AoU data, focusing on tissue associations and demographic impacts, validated by NIH All of Us cohort.	MS Bioinformatics	IUPUI, Indianapolis, IN
Intern 14	Reviewed 15 RNA modification databases to analyze and interpret their significance for future molecular biology research.	BS Biology	Barry University, Miami Shores, FL

**Table 2:** Sentiment Analysis Results for Program Satisfaction, Skills Confidence, Valuable Learning Opportunities, Content and Structure Alignment, and Professional Competence Development

Category	Min	1st Qu.	Median	Mean	3rd Qu.	Max

Overall Program Satisfaction	−0.45	0.075	1.100	2.205	2.200	10.100
Confidence About Skills Gained	0.000	0.800	1.100	0.9909	1.100	2.200
Experienced Valuable Learning Opportunities	0.000	0.800	1.100	1.073	1.100	3.100
Program’s Content Aligned with Career Goals	−0.50	0.250	0.600	0.5545	1.100	1.100
Developed Professional Competence	0.000	0.500	0.500	0.8545	1.100	2.500

## Data Availability

Due to the limited sample size, releasing de-identified information raises ethical concerns and risks of reidentification. Therefore, it is not feasible.

## List of Abbreviations:

NLM: National Library of Medicine
NIH: National Insititues of Health
IU-MSI: Indiana University and Minority Serving Institutions
STEM: Science, Technology, Engineering, Mathematics
HBCU: Historically Black college or University
AI: Artificial Intelligence
RNA: Ribonucleic acid
ICU: Intensive Care Unit
IUPUI: Indiana University-Purdue University, Indianapolis
UNIPROT: The Universal Protein Resource
MS: Master of Science
BS: Bachelor of Science
